# Modulation of p53 Expression Using Antisense Oligonucleotides Complementary to the 5′-Terminal Region of p53 mRNA In Vitro and in the Living Cells

**DOI:** 10.1371/journal.pone.0078863

**Published:** 2013-11-11

**Authors:** Agnieszka Gorska, Agata Swiatkowska, Mariola Dutkiewicz, Jerzy Ciesiolka

**Affiliations:** Institute of Bioorganic Chemistry, Polish Academy of Sciences, Poznan, Poland; Imperial College London, United Kingdom

## Abstract

The p53 protein is a key player in cell response to stress events and cancer prevention. However, up-regulation of p53 that occurs during radiotherapy of some tumours results in radio-resistance of targeted cells. Recently, antisense oligonucleotides have been used to reduce the p53 level in tumour cells which facilitates their radiation-induced apoptosis. Here we describe the rational design of antisense oligomers directed against the 5′-terminal region of p53 mRNA aimed to inhibit the synthesis of p53 protein and its ΔNp53 isoform. A comprehensive analysis of the sites accessible to oligomer hybridization in this mRNA region was performed. Subsequently, translation efficiency from the initiation codons for both proteins in the presence of selected oligomers was determined in rabbit reticulocyte lysate and in MCF-7 cells. The antisense oligomers with 2′-*O*Me and LNA modifications were used to study the mechanism of their impact on translation. It turned out that the remaining RNase H activity of the lysate contributed to modulation of protein synthesis efficiency which was observed in the presence of antisense oligomers. A possibility of changing the ratio of the newly synthetized p53 and ΔNp53 in a controlled manner was revealed which is potentially very attractive considering the relationship between the functioning of these two proteins. Selected antisense oligonucleotides which were designed based on accessibility mapping of the 5′-terminal region of p53 mRNA were able to significantly reduce the level of p53 protein in MCF-7 cells. One of these oligomers might be used in the future as a support treatment in anticancer therapy.

## Introduction

The p53 is one of the transcriptional factors which prevents cancer development *via* cell cycle arrest and induction of apoptosis [1]–[4]. The p53 protein is a crucial player in maintaining cell homeostasis, and the p53 regulation undergoes rigorous control. In normal conditions p53 protein is ubiquitinated by Mdm2/Hdm2 and subsequently it is degraded by proteosome. In response to stress events such as ionizing radiation or genotoxic stress the p53 is phosphorylated to prevent the interaction with Mdm2. Consequently, the level of p53 protein is increasing and p53-response reactions are triggered [5], [6].

Besides the full-length p53 protein several isoforms have been identified in the cell [7]–[9]. The p53 isoforms are expressed differently depending on cell type, cell cycle stages and stress conditions [10]. The major p53 isoform is ΔNp53 which lacks 39 amino acids corresponding to the TAD domain with the Mdm2 binding site [11]–[13]. The ΔNp53 has been proposed to be one of the major regulators of the level of the full-length p53 protein [5], [12], [13]. Translation initiation codons for both p53 and ΔNp53 are located in the 5′-terminal region of p53 mRNA. This region also contains an IRES element (Internal Ribosome Entry Site) which drives the translation initiation process mostly under stress conditions [12], [14], [15]. Recently, a secondary structure model of the 5′-terminal region of p53 mRNA has been proposed which greatly facilitates detailed studies on the functioning of this region [16], [17]. Importantly, it has been shown that in the model mRNA constructs the 5′ non-coding region should include a part of the p53 coding sequence up to the second initiation codon for ΔNp53 to mimic the structure of this region in the full-length p53 mRNA [16].

Ionizing radiation is still one of the most effective forms of anti-cancer therapy. However, cell radioresistance is observed in some malignant tumours [18]. It has been shown that p53 response upon IR-induced DNA damage leads to the activation of numerous transcription factors, such as p21^WAF1/CIP1^, GADD45 and cyclins, and subsequently to cell arrest and/or apoptosis [19], [20]. The radioresistance mechanism seems to be correlated with the balance between the function of p53 as a cell apoptotic activator and p53-dependent cell arrest. It has been observed that the activation of p53 upon ionizing radiation induces longer arrest at G2 checkpoint in the cells with wild-type p53 and to some extent it may influence radiosensitivity. However, the association of the role of p53 with radiation-induced cell arrest is still not fully understood. Nevertheless, it has been shown that inactivation of both p53 and p21 by antisense oligonucleotides in non-small lung cancer cells (NSCLC) results in the reduction of G2 arrest and an increase in radiation-induced apoptosis [21]. Moreover, inhibition of p21 using the antisense strategy results in abrogating the G1 arrest and at the same time enhancing the radiosensitivity of cancer cells [22], [23].

The antisense oligonucleotide strategy has been successfully used for decades to inhibit gene expression *via* RNase H-dependent and independent mechanisms [24]–[28]. Using antisense oligomers to reduce the expression of p53 protein in cancer cells as a support treatment in radiotherapy seems to be an appealing idea considering the promising results which have been earlier reported [21]. However, in order to achieve that goal it is important to design oligomer tools that will work with high effectiveness and specificity. Despite the straightforward concept of antisense strategy many factors influencing the oligomer-target interaction, including among others the structural features of the RNA target, its accessibility to oligomer hybridization, resistance of antisense oligomers to degradation, and the mechanism of their action on the RNA target, must be considered to obtain satisfactory results [29].

In our study the rational design of antisense oligonucleotides targeting the 5′-terminal region of p53 mRNA was used. Based on the comprehensive analysis of the sites accessible to oligomer hybridization in a model mRNA transcript several antisense compounds were designed. Modulation of protein translation by these antisense oligonucleotides was tested in rabbit reticulocyte lysate. To gain more details about the mechanisms which are responsible for changes in the synthesis of the full-length p53 and ΔNp53 isoform antisense oligonucleotides bearing different chemical modifications were also used. Subsequently, selected oligomers were applied in MCF-7 cells to verify their potential as regulators of p53 protein expression.

## Materials and Methods

### DNA Template Construct and Antisense Oligonucleotides

The DNA template for the 5′-terminal region of p53 mRNA, ΔNp53utr, was amplified from human liver cDNA (Ambion) using the following forward (F) and reverse (R) primers: 5′-CTAGAGCCACCGTCCAGGGAGC-3′ complementary to exon 1, and 5′-TCCATTGCTTGGGACGGCAAGG-3′ complementary to exon 4. The restriction sites were then added using forward primer FXba 5′-AAGTCTAGAGCCACCGTCCAGG-3′ bearing restriction site for XbaI and reverse primer RCsp 5′-TCCATTGCTTGGGACGGCAAGG-3′ with Csp45I restriction site. The PCR reaction mixture contained: 0.5–20 ng of cDNA (or dsDNA) template, 0.5 µM of each forward and reverse primers, 75 mM Tris-HCl pH 8.8, 20 mM (NH_4_)_2_SO_4_, 0.01% Tween 20, 200 µM each dNTP, 1.5 mM MgCl_2_ and 30 U/ml Taq polymerase (Fermentas). The reaction products were purified by phenol/chloroform (1∶1, v/v) extraction and precipitated with ethanol or purified using DNA Purification System (Promega). The obtained ΔNp53utr DNA insert was cloned into the pRL-CMV vector (Promega) between T7 promoter and *Renilla* luciferase coding sequence. The sequence of the construct was confirmed by sequencing.

Primers and unmodified antisense oligonucleotides were synthesized by Oligo Service IBB PAS, Warsaw. 2′-*O*Me oligomer no. 1 and control oligomer were synthesized by Future Synthesis, Poznan. 2′-*O*Me oligomer no. 7b was a gift from prof. R. Kierzek. All GAP oligomers were synthesized by Exiqon.

### In vitro Transcription

For *in vitro* transcription the plasmid construct was linearized using NotI restriction enzyme. *In vitro* transcription reaction with AmpliScribe T7, T3 and SP6 High Yield Transcription Kit (Epicentre Biotechnologies) or RiboMax Large Scale RNA Production System-T7 (Promega) was performed as recommended by the manufacturers. The 5′-capped ΔNp53utr-Luc RNA was synthesized in the presence of 3 mM Anti-Reverse Cap Analogue (ARCA, Epicentre Biotechnologies), 1.5 mM GTP and 7.5 mM of the remaining nucleotide triphosphates. After transcription reaction 1 unit of DNase I was added and the reaction was incubated for 15 min at 37°C. The RNA was purified using RNeasy MinElute Cleanup kit (Qiagen). The size and integrity of RNA transcript was verified using agarose gel electrophoresis and ethidium bromide staining.

### Mapping of RNA Accessibility to Hybridization with DNA 6-mer Libraries and RNase H Digestion

Mapping of accessible sites in RNA transcripts was performed as described previously [30]. Briefly, prior to digestion with *E. coli* RNase H (Fermentas) the 0.5 pmol of ΔNp53utr-Luc RNA was renatured in buffer containing: 40 mM Tris-HCl pH 8.0, 40 mM KCl, 10 mM MgCl_2_, 1 mM DTT, 0.1 mM EDTA by heating for 2 min at 65°C and slow cooling to 37°C. RNase H was added to the final concentration of 250 units/ml. The cleavage reactions were induced by adding separately four DNA 6-mer libraries (final concentration of 200 µM) to four RNA samples (in 10 µl final reaction volume). The reaction mixtures were incubated for 10 and 30 min at 37°C. The reactions were stopped by adding an equal volume of 20 mM EDTA. The reaction products were purified by phenol/chloroform (1∶1, v/v) extraction and precipitated with 0.3 M sodium acetate pH 5.2, 1 µl of glycogen (20 mg/ml) and 3 volumes of ethanol at −20°C for one hour.

### DMS Probing in Rabbit Reticulocyte Lysate, RRL

Ten pmol of ΔNp53utr-Luc RNA in 100 mM KCl was renatured for 3 min at 90°C and slowly cooled (0.1°C/sec) to 4°C. The RNA solution was supplemented with MgCl_2_ to adjust to 0.5 mM final concentration of Mg^2+^ ions and the mixture was incubated for 10 min at 37°C. Subsequently, the RNA solution was added to 17.5 µl of RRL to a final volume of 23.75 µl and the mixture was incubated for another 10 min at 30°C. Finally, 1.25 µl of 5% DMS in ethanol (0.25% final concentration) was added except the control reaction. All reactions were incubated for 2 min at 30°C. Modification with DMS was stopped by 10% ice-cold ß-mercaptoethanol. Immediately, the RNA was isolated using 300 µl of TriReagent (Molecular Research Centre, Inc.) according to the manufacturer protocol.

### Primer Extension

To determine RNase H cleavage sites primer extension reaction was performed with 5′-end-[^32^P]-labelled primer 5′-TAATAAATGAATCAAGAACATCC-3′ which was complementary to nucleotides 81–103 in *Renilla* luciferase coding sequence. The reaction mixtures containing: RNA (0.5–1 pmol), DNA primer (2 pmol) in a final volume of 12 µl were incubated at 90°C for 1 min and placed on ice for 10 min. Subsequently, 8 µl of reverse transcription mix was added and the final reaction conditions were as follows: 50 mM Tris-HCl pH 8.3, 50 mM KCl, 4 mM MgCl_2_, 10 mM DTT, 500 µM each dNTP and 100 units of RevertAid™ M-MuLV reverse transcriptase (Fermentas). The reaction was incubated at 42°C for 40 min. The sequencing ladders were generated in the same way and the final concentration of each ddNTP was 0.2 mM. The obtained cDNAs were treated with 1 µl of 4 M NaOH, heated at 95°C for 5 min, placed on ice and mixed with 160 mM Trizma. Then cDNAs were precipitated with 0.3 M sodium acetate pH 5.2, 1 µl of glycogen (20 mg/ml) and 3 volumes of ethanol. The samples were heated at 95°C for 2 min in 8 M urea/dyes/20 mM EDTA solution and loaded onto 8% acrylamide, 0.75% bis-acrylamide and 7 M urea gels. The separated products were visualized using FLA 5100 image analyzer (Fuji).

To analyze DMS modifications reverse transcription reactions were prepared with 5′-end-florescent-labelled primer 5′-TAATAAATGAATCAAGAACATCC-3′. The primer was labelled with one of dyes: VIC, 6-FAM, PET or NED (Applied Biosystems). The RNA samples after modification were subjected to reverse transcription reaction with the primer labelled with VIC. The control reaction with unmodified RNA was prepared using the primer labelled with 6-FAM. The primer used for sequencing was labelled with PET (for sequencing with ddT) and NED (for sequencing with ddA or ddG). The primer extension mixtures were prepared as described above using the following final reaction conditions: 50 mM Tris-HCl pH 8.3, 75 mM KCl, 3 mM MgCl_2_, 6 mM DTT, 500 µM each dNTP and 50 units of SuperScript™ III reverse transcriptase (Invitrogen). The reactions were incubated at 45°C for 1 min, 52°C for 1 hour and 65°C for 5 min. The cDNA fragments from each of the four reverse transcription reactions were combined and resolved on Applied Biosystems 3130 x l capillary electrophoresis DNA sequencing apparatus. Raw data were analyzed by ShapeFinder software [31].

### Translation in the Presence of Antisense Oligonucleotides in RRL

Translation reactions were performed in nuclease-treated rabbit reticulocyte lysate (RRL) as described by the manufacturer (Promega). 2.5 pmol of 5′-capped ΔNp53utr-Luc RNA in a volume of 5.5 µl was denatured at 65°C for 3 min and immediately placed on ice for 5 min. The RNA solution was added to 19.5 µl of the translation mixture containing: 17.5 µl of RRL, 20 µM amino acid mix without methionine, 1 µl of [^35^S]-methionine (1000 Ci/mol) (Hartman Analytic) and 20 units of recombinant ribonuclease inhibitor (Promega). The reaction mixture was incubated for 2 min at 30°C. Subsequently, antisense oligonucleotides were added to the final concentration of 0.5 or 2 µM and the reactions proceeded at 30°C for 90 min. A control reaction was performed without oligonucleotides. After incubation, RNase A was added to a final concentration of 0.2 mg/ml and the samples were incubated for 5 min at room temperature. Translation reaction products were resolved on 15% SDS-polyacrylamide gels, followed by radioisotope imaging with FLA 5100 image analyzer (Fuji). Band intensities were analyzed using MultiGauge software (Fuji).

### RNase H Assay in RRL

2.5 pmol of 5′-end-[^32^P]-labelled ΔNp53utr-Luc RNA was heated at 65°C for 2 min and chilled on ice for 5 min. The RNA was combined with 17.5 µl of RRL and the mixture was incubated for 5 min at 30°C. Then, the antisense oligonucleotide no. 1 or no. 7b was added to the final concentration of 2 µM. After 30 min incubation at 30°C all reactions were immediately placed on ice. RNAs were isolated using TriReagent as described previously.

### Cell Culture and Antisense Oligonucleotide Transfection

MCF-7 cells were maintained in DMEM supplemented with 10% fetal bovine serum (FBS), non-essential amino acids (Gibco-BRL), 100 U/ml of penicillin G, 0.1 mg/ml streptomycin sulphate and 0.25 µg/ml amphotericin B (Antibiotic Antimycotic – Sigma) at 37°C in 5% carbon dioxide atmosphere. Cells between 4 and 15 passages were used for transfection. Transfection was performed when cell confluence reached 50–80%. Antisense oligonucleotides with the final concentration of 1 µM (0.5, 0.25, 0.15 and 0.1 µM of GAP oligomer no. 7b) were transfected into cells using Lipofectamine 2000 or Lipofectamine RNAiMax according to a transfection protocol (Invitrogen). Four hours post transfection cells were washed in PBS and fresh medium was supplied. Then cells were washed in PBS and lysed at each time point, respectively. Prior to antisense oligonucleotide transfection experiments the transfection assay was performed using 5′-Fam-conjugated DNA oligomer (5′-Fam-ACCAGGGCGTATCTCTCCATA-3, the nucleotide sequence of control oligomer which was used in *in vivo* experiments) to confirm that the oligomers enter to MCF-7 cells (data not shown).

### Western Blot, RNA Isolation and RT-PCR

For western blot MCF-7 cell lysates were prepared in the buffer: 62.5 mM Tris-HCl pH 6.8, 2% SDS, 10% glycerol and 50 mM DTT. Total cell lysates were incubated for 5 min at 95°C and then loaded on 10% SDS-PAGE gel and proteins were transferred to a nitrocellulose membrane. The blot was probed with mouse monoclonal antibody p53 (Pab 1801) and GAPDH (Santa Cruz Biotechnology). Primary antibody was detected by Goat Anti-Mouse-HRP (Thermo Scientific Pierce) and visualization was performed by using enhanced chemiluminescent visualization (ECL) system (Thermo Scientific Pierce).

For RT-PCR after cells transfection with antisense oligonucleotides total RNA was isolated from MCF-7 cells using TriReagent (Molecular Research Centre, Inc.) according to the manufacturer protocol. First strand cDNA was synthesized from 300 ng of RNA using 100 ng of oligo(dT)_18_ primer and 100 units of SuperScript™ III reverse transcriptase (Invitrogen). cDNA was treated with 250 units/ml of RNase H (Fermentas) for 20 min at 37°C. Equal volumes of cDNA were used to amplify p53 and β-actin using following primers: p53 F, 5′-CTAGAGCCACCGTCCAGGGGAGC-3′, p53 R, 5′-GTCTTGGCCAGTTGGCAAAACATC-3′, β-actin F, 5′-AGAGCAAGAGAGGCATCCTG-3′, β-actin R, 5′-CGACGTAGCACAGCTTCTCC-3′. PCR reactions for p53 and β-actin were performed in the following conditions: 30 cycles of denaturing at 95°C for 30 s, annealing at 62°C (for p53) and 60°C (for β-actin) for 30 s and extending 72°C for 30 s. The obtained products were resolved on 1% agarose gels.

## Results

### Sites Accessible to Oligomer Hybridization in the 5′-terminal Region of p53 mRNA

**Figure 1:**
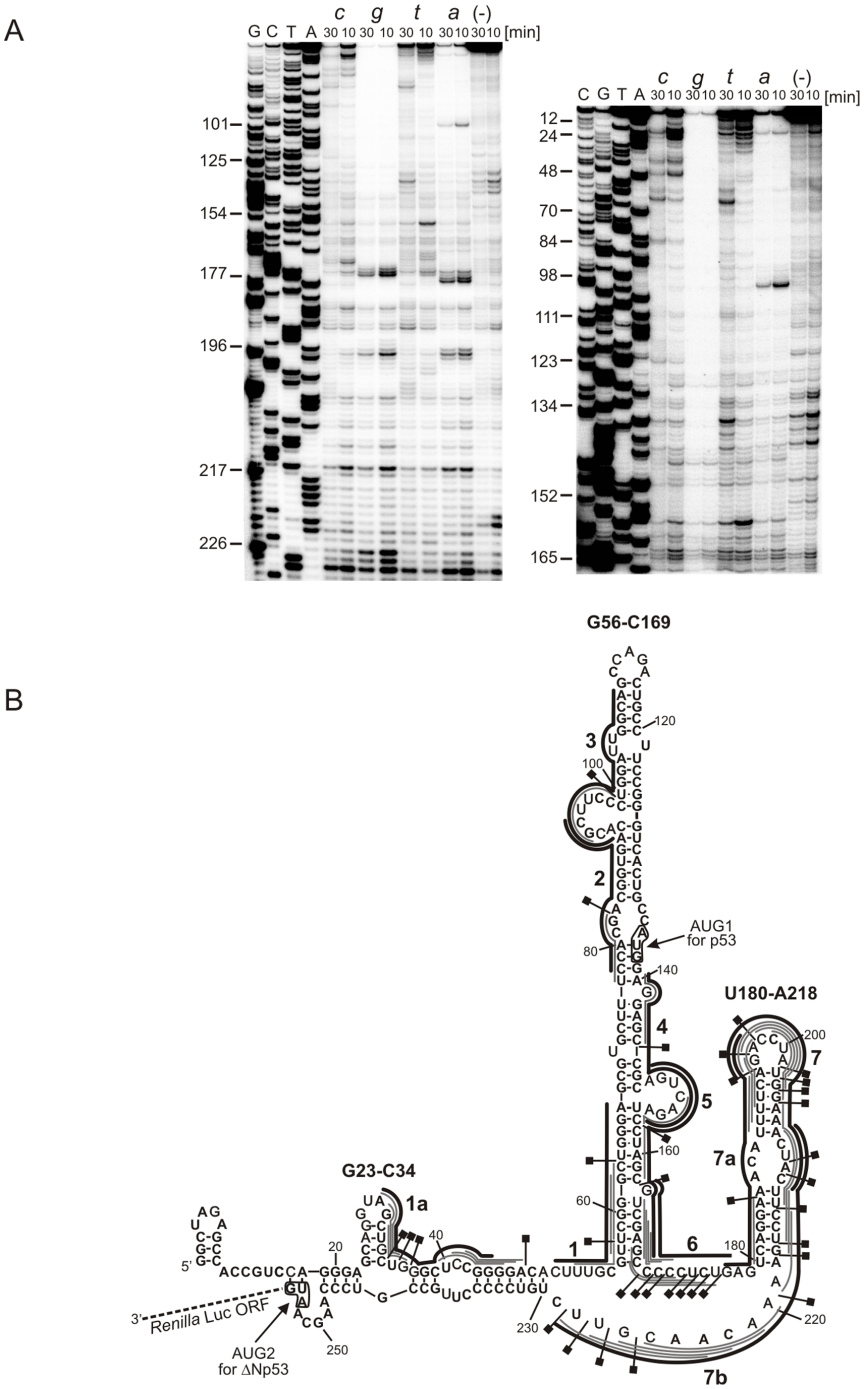
Mapping of the accessibility of the 5′-terminal region of ΔNp53utr-Luc transcript to oligonucleotide hybridization. Semi-random libraries of DNA 6-mers and RNase H hydrolysis of RNA-DNA hybrids were used to search for sites accessible to hybridization in the 5′-terminal region of ΔNp53utr-Luc transcript. (**A**) The cleavage sites were identified by reverse transcription with the 5′-end-[^32^P]-labelled DNA primer that was bound to nucleotides 232–257 of the ΔNp53utr-Luc sequence. The cDNA products were analyzed on 8% polyacrylamide gels. Selected cytosine and guanosine residues are marked on the left and the short and long run of the gel is shown. Lane (−) denotes the control reaction in the absence of semi-random oligonucleotide libraries. A, T, C, G – sequencing reaction with adenosine, thymidine, cytosine and guanosine dideoxy terminating nucleotides, respectively. (**B**) The cleavage sites occurring in the presence of libraries *a*, *c*, *g*, *t* displayed on the RNA secondary structure model. Continuous grey lines along the RNA sequence show the most probable positions of oligonucleotide hybridization on the ΔNp53utr-Luc transcript. The designed specific antisense oligonucleotides are marked with black lines and numbered respectively.

A model mRNA construct, ΔNp53utr-Luc, was synthetized in which the sequence corresponding to the 5′-terminal region of p53 mRNA extended to the initiation codon for ΔNp53 isoform was attached to the sequence encoding the reporter protein, *Renilla* luciferase (Fig. 1). For mapping the sites accessible to oligomer hybridization 6-mers semi-random DNA libraries and RNase H hydrolysis were used [30]. This approach has been shown to be particularly useful in screening for target sites for antisense oligonucleotides in highly structured RNA molecules. For example, the accessibility to oligonucleotide hybridization of region X of hepatitis C virus [32] and the genomic and antigenomic HDV ribozymes have been characterized using that method [30].

The sites accessible to hybridization in the 5′-terminal region of ΔNp53utr-Luc transcript were mapped based on RNase H cleavages which occurred in the presence of the 6-mer DNA libraries (Fig. 1). The third nucleotide from the 5′ end of each oligomer was fixed to facilitate the correlation of cleavage sites with the predicted positions of hybridizing oligonucleotides [30]. Mostly, antisense oligomers were mapped to the U180-A218 hairpin. Especially, the apical loop and the 3′ side of the hairpin were highly accessible (Fig. 1B). RNase H cleavages also occurred in the hinge region between the helixes G56–C169 and U180-A218. The 5′-proximal part of ΔNp53utr-Luc was less accessible and only the 3′ side of the small hairpin G23-C34 and an adjacent region provided access to oligonucleotides. The G56-C169 hairpin, which has been shown to be a characteristic structural element of the 5′-terminal region of p53 mRNA, was poorly mapped by RNase H and DNA libraries. A few oligomers were, however, able to bind to the structural distortions located along the hairpin stem. Unexpectedly, an apical loop of the helix and the downstream region extending up to the AUG1 initiation codon were completely inaccessible to hybridization. It has been shown that the mapping approach based on DNA libraries and RNase H digestion allows finding regions which are accessible to hybridization despite their double-stranded character. In such cases, oligomer binding is usually facilitated by the presence of weak base pairs and structural distortions in double-stranded regions of the RNA target [30], [32]. This is nicely illustrated by the unexpectedly high accessibility to hybridization of the U180–A218 hairpin in which several A–U base pairs and an asymmetric internal loop are present (Fig. 1B). A similar effect is also observed in the bottom part of helix G56–C169 where three G–U wobble pairs and a single-nucleotide bulge weaken the double-stranded steam which in turn becomes accessible to hybridization.

### Secondary Structure of the 5′-terminal Region of p53 mRNA is Preserved in ΔNp53utr-Luc Transcript

The secondary structure of the 5′-terminal region of p53 mRNA has been recently determined *in vitro* [16], [17]. Since antisense oligomers were planned to be used in rabbit reticulocyte lysate (RRL), and further in the living cell, we decided to perform a structural analysis of the 5′-terminal region of ΔNp53utr-Luc transcript in conditions of the translation system. Moreover, we wanted to be sure that the presence of the sequence encoding the reporter protein does not affect the folding of the RNA fragment corresponding to the 5′-terminal region of p53 mRNA.

**Figure 2:**
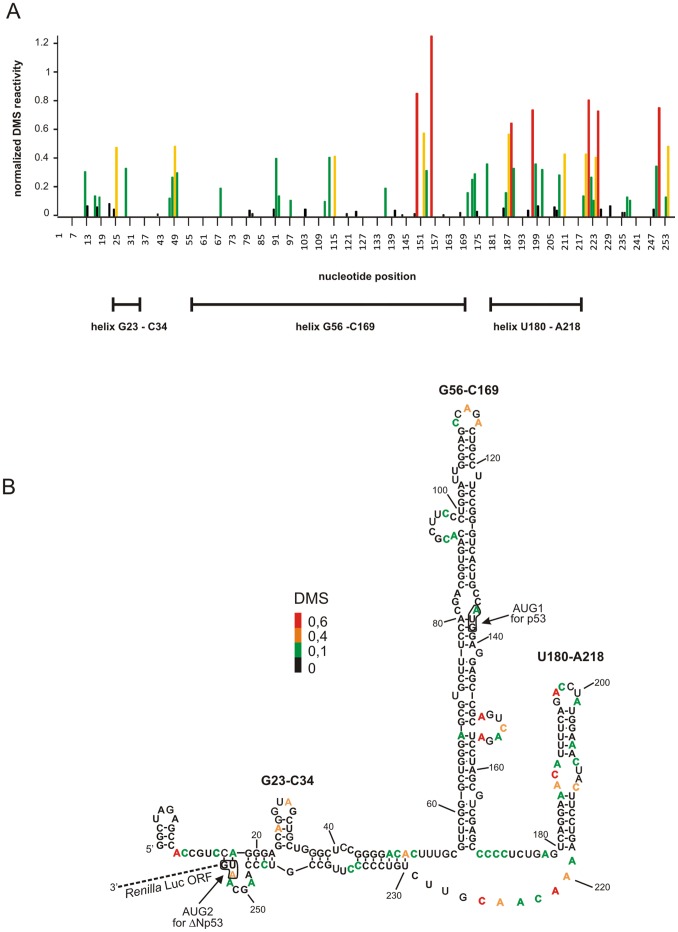
Structure probing of the 5′-terminal region of p53 mRNA in a model RNA construct in rabbit reticulocyte lysate using DMS. (**A**) Bars represent normalized DMS reactivity as a function of nucleotide position. Bars are coloured using the scheme shown in panel B. (**B**) The secondary structure of ΔNp53utr in the model RNA construct containing *Renilla* luciferase coding sequence. Nucleotides modified by DMS are coloured according to their reactivity.

The structure of the ΔNp53utr-Luc transcript was probed *in vitro* using Pb^2+^-induced cleavage [33], [34], SHAPE, and chemical modification method with DMS (Fig. S1). The results revealed no changes in folding of the 5′ part of the transcript in comparison with the 5′-terminal region of the full-length p53 mRNA [16]. Then, the structure of this region was determined in RRL. It turned out that DMS was particularly suitable for probing the RNA structure in the lysate. As shown in Figure 2 the modification sites are in good agreement with the earlier proposed secondary structure model of the 5′-terminal region of p53 mRNA [16]. Modified A and C residues are located in the bulges and apical loops of hairpins G56-C169 and U180-A218, and in the small hairpin G23-C34. Additionally, the nucleotide stretches spanning positions 170–180 and 219–229 were fully accessible to chemical modification with DMS. This confirms the lack of stable structural elements in these regions. Therefore, we concluded that folding of the 5′-terminal part of ΔNp53utr-Luc in RRL is similar to that proposed for the corresponding region of the full-length p53 mRNA. Further, we envisage that the same folding of the 5′-terminal region of p53 mRNA is likely preserved in endogenous p53 mRNA in the living cell.

### Modulation of Protein Synthesis Occurring from the Initiation Codons for p53 and ΔNp53 by Antisense Oligonucleotides in RRL

Based on the results of accessibility mapping several antisense DNA oligomers were designed to the 5′-terminal region of p53 mRNA which are listed in Table 1. While designing the oligomers, the access to hybridization and the overall ΔG value reflecting oligomer-target binding energy were taken into account. Applying the OligoWalk function in RNAStructure 5.2 program [35] all oligomers were designed in a way to exhibit similar ΔG values (Table 1). Subsequently, the designed oligomers were tested in the RRL translation system to monitor whether they are able to modulate protein expression from the initiation codons for p53 and/or ΔNp53.

**Table 1:** Antisense DNA oligomers.

Oligomer	Length [nt]	Sequence (5′–3′)	Target site in ΔNp53utr RNA (5′–3′)	ΔG_overall_ [kcal/mol]
1a	16	CCGGAGCCCAGCAGCT	28–44	−8.5
1	19	TCCCAGCCCGAACGCAAAG	50–68	−8.5
2	17	GCGTGTCACCGTCGTGG	78–94	−8.8
3	18	CTGCCAATCCAGGGAAGC	93–110	−8.7
4	17	TCCTCGGCGTCAGTCTA	140–156	−8.5
5	21	TCAGTCTAGGATCGCAGCTCG	150–169	−8.5
6	15	CAGAGGGGGCTCGAC	163–177	−8.5
7a	21	TCTGAAAATGTTTCCTGACTC	177–197	−8.3
7	19	GAAGTAGTTTCCATAGGTC	186–214	−9.7
7b	21	AACGTTGTTTTCAGGAAGTAG	208–228	−7.5

**Figure 3:**
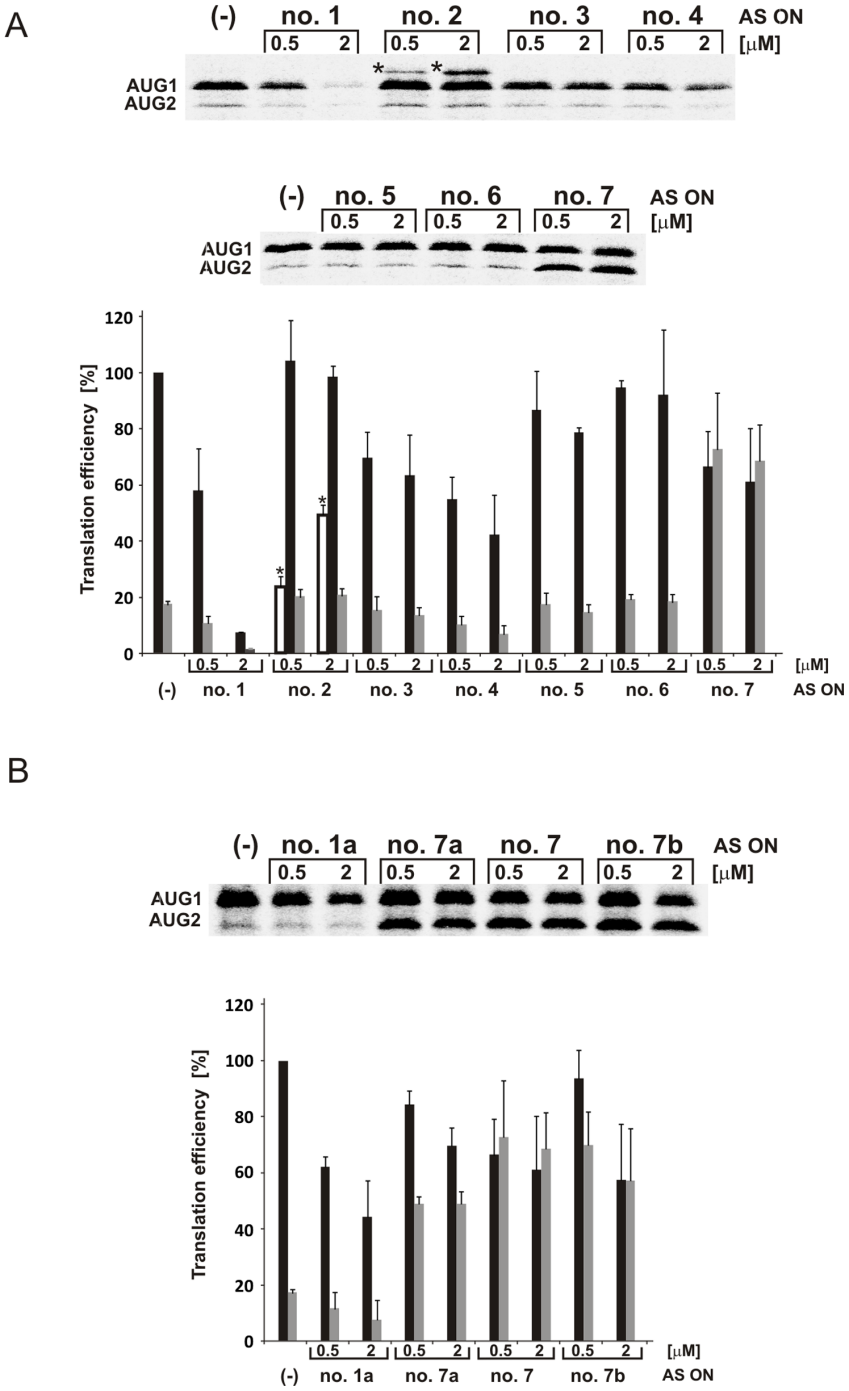
The effects of antisense oligonucleotides on translation initiated from AUG1 and AUG2 codons localized in ΔNp53utr RNA. The RNA transcript ΔNp53utr-Luc containing a cap analogue at the 5′ end was translated in RRL in the presence of [^35^S]-methionine and with 0.5 and 2 µM concentration of antisense oligonucleotides targeted to ΔNp53utr RNA. Translation in the presence of oligomers no. 1–7 (**A**) and oligomers no. 1a, 7, 7a and 7b (**B**). The products were resolved on SDS-polyacrylamide gels. In the case of translation reaction in the presence of antisense oligomer no. 2, an additional band, which probably results from translation initiated from a non-AUG codon, was labelled by an asterisk. Quantitative evaluation of the gels is shown on the bar charts. Translation from AUG1 is denoted by black bars, from AUG2 by grey bars and from non-AUG start site by white bars. All values are means of at least three independent experiments and they were normalized to the values with no antisense oligonucleotide present (−).

Translation reactions were performed with 5′-capped ΔNp53utr-Luc RNA transcript in the presence of each of the tested antisense oligonucleotides. As shown on the autoradiogram in Figure 3A two protein products were synthetized in all cases. The upper band on the gel corresponds to the fusion protein comprising the full-length reporter protein and 39 amino acids from the N-terminus of p53. The fusion protein is translated from the first initiation codon AUG1 for p53 which is embedded into the G56-C169 hairpin (Fig. 1). The lower band represents the full-length *Renilla* luciferase which is synthetized from the second initiation codon AUG2 for ΔNp53 isoform. In the case of oligomer no. 2, an additional, slower migrating band appears on the gel. The observed protein product results presumably from the usage of a non-standard initiation codon. However, the nature and origin of this product was not investigated further.

It turned out that two out of seven tested antisense oligonucleotides remarkably changed the translation level from one or both initiation codons. In the presence of oligomer no. 1 complementary to the bottom part of hairpin G56-C169, a reduced translation level was observed from both AUG1 and AUG2 codons (Fig. 3A). The oligomer used at 2 µM concentration diminished protein synthesis from AUG1 approximately 5-fold compared with the control level. Since only translation from AUG1 was strongly affected in the presence of a higher concentration of the oligomer, the ratio of the synthesized proteins was twice lower than in the control reaction.

In the presence of oligomer no. 7 complementary to the apical loop and partially to the 3′ side of the U180-A218 hairpin the translation efficiency from AUG1 decreased only slightly even at 2 µM oligomer concentration. Surprisingly, protein synthesis from AUG2 increased approximately 3-fold compared to the control value (Fig. 3A). However, higher concentration of the oligomer did not enhance the observed effect. Strong induction of the protein synthesis only from the second initiation codon resulted in a large change of the balance between the proteins translated from AUG1 and AUG2. Both proteins were synthetized in a ratio of approximately 1∶1 (Fig. 3A).

Since two oligomers (no. 1 and no. 7), which hybridize to the functionally important elements of the 5′-terminal region of p53 mRNA, induced significant changes in protein synthesis we decided to design a few more antisense oligonucleotides. They covered the surroundings of the binding sites of oligomers no. 1 and no. 7 (Table 1, oligomers no. 1a, 7a and 7b). In the presence of oligomer no. 1a, which was complementary to the region A20-G44 the translation level was slightly decreased, mostly from codon AUG1. However, in comparison with the observed effect for oligomer no. 1, protein synthesis did not change strongly. Variants no. 7a and no. 7b, which were complementary to the hairpin U180-A218 and to the nucleotide stretch A219–U228 enhanced the translation from AUG2 at a similar level as was observed for oligomer no. 7 (Fig. 3B).

### RNase H-dependent and Independent Mechanisms of Modulation of Protein Expression by Antisense Oligonucleotides in RRL

Since the impact of antisense oligonucleotides on translation was tested in cell-free conditions in RRL we wanted to check whether the specific composition of the lysate might influence the observed effects. It has been previously reported that the remaining RNase H activity could be present in cell-free translation systems such as wheat germ extract and rabbit reticulocyte lysate [36], [37]. In order to find out whether the remaining RNase H activity contributed to the translation results observed in the presence of oligomers no. 1 and no. 7b their derivatives with all 2′-*O*-methyl residues (2′-*O*Me) and LNA/phosphorothioates – gapmers (GAP) were designed (Table 2). It is known that antisense oligonucleotides bearing methyl groups at 2′ position of all ribose residues do not activate RNase H-mediated cleavage of RNA target whereas LNA/PS gapmers with contiguous stretches of at least six deoxyribonucleotides can support RNase H activity [38], [39].

**Table 2:** Modified antisense oligomers.

Oligomers	Length [nt]	Sequence (5′-3′)	Target site in ΔNp53utr-Luc RNA (5′-3′)
2′-*O*Me 1	19	*TCCCAGCCCGAACGCAAAG*	28–44
2′-*O*Me 7b	21	*AACGTTGTTTTCAGGAAGTAG*	208–228
2′-*O*Me (+)	21	*ACCAGGGCGTATCTCTCCATA*	–
GAP 1	19	T_L_C_L_C_L_C_s_A_s_G_s_C_s_C_s_C_s_G_s_A_s_A_s_C_s_G_s_C_s_A_s_A_L_A_L_G_L_	28–44
GAP 7b	21	A_L_A_L_C_L_G_s_T_s_T_s_G_s_T_s_T_s_T_s_T_s_C_s_A_s_G_s_G_s_A_s_A_s_G_s_T_L_A_L_G_L_	208–228
GAP (+)	21	A_L_C_L_C_L_A_s_G_s_G_s_G_s_C_s_G_s_T_s_A_s_T_s_C_s_T_s_C_s_T_s_C_s_C_s_A_L_T_L_A_L_	–

In oligomer sequences: italic letters –2′-*O*-methyl monomers, N_L_ – LNA, N_s_ – phosphorothioates; (+) denotes antisense oligonucleotides complementary to *Firefly* luciferase coding sequence.

**Figure 4:**
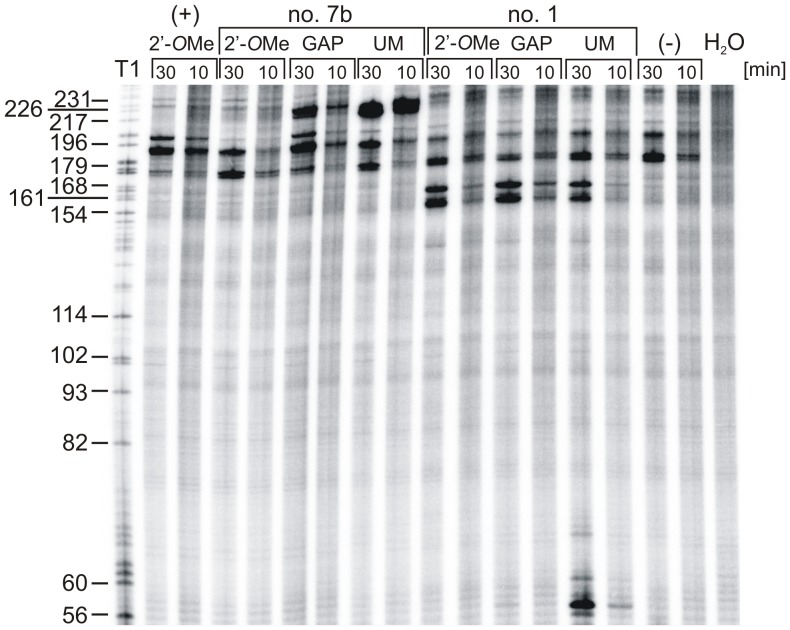
RNase H assay in rabbit reticulocyte lysate in the presence of antisense oligomers no. 1 and 7b targeted to ΔNp53utr RNA. The 5′-end-[^32^P]-labelled ΔNp53utr-Luc RNA was incubated in RRL and subsequently antisense oligomers no. 1 and no. 7b in their unmodified (UM) and modified (2′-*O*Me or GAP) form were added to the mixture. After 10 and 30 min incubation at 30°C, RNA was isolated and resolved on 8% polyacrylamide gel in denaturing conditions. The ΔNp53utr-Luc RNA was also subjected to limited hydrolysis by RNase T1 in denaturing conditions to determine the positions of RNase H cleavages. Lanes (−) and H_2_O indicate control reactions in the absence of antisense nucleotide in RRL and water, respectively. Lane (+) denotes the reaction in the presence of a control antisense oligonucleotide which is complementary to the *Firefly* luciferase sequence.

A radioactively labelled ΔNp53utr-Luc transcript was incubated with each unmodified or modified antisense oligonucleotide in RRL. In the presence of unmodified oligomer no. 7b and its GAP variant characteristic RNase H cleavages occurred at the predicted sites, in the region G217-G226 (Fig. 4). The 2′-*O*Me variant did not activate RNase H, as it was expected. In the case of unmodified oligomer no. 1, cleavages appeared in the region between G56 and G60. However, surprisingly, GAP oligomer no. 1 did not trigger RNase H activity. Moreover, all variants of oligomer no. 1 induced cleavages in the region between G161 and G168 which is opposite to their hybridization site in the RNA secondary structure (Fig. 4). To explain this observation, an RNase H assay was performed in the presence of unmodified oligomer no. 1 and its GAP variant using a commercially available enzyme from *E. coli* (Fig. S2). Both oligomers supported RNase H activity and the predicted cleavage patterns were obtained. However, there were no additional cleavages in the region G161-G168, which were observed in RRL (Fig. 2). This discrepancy might result from a slightly different activity of eukaryotic RNase H which was present in RRL and *E. coli* RNase H which was used in the test assay [40]. According to an alternative explanation, it might be suspected that binding of each variant of oligomer no.1 to the C50-A68 region resulted in partial unwinding of the double-stranded region at the bottom of hairpin G56-C169. The RNA strand located opposite to the oligomer hybridization site became more flexible. In consequence, it was susceptible to spontaneous breakage or cleavage by some other RNases which might be remaining in RRL.

**Figure 5:**
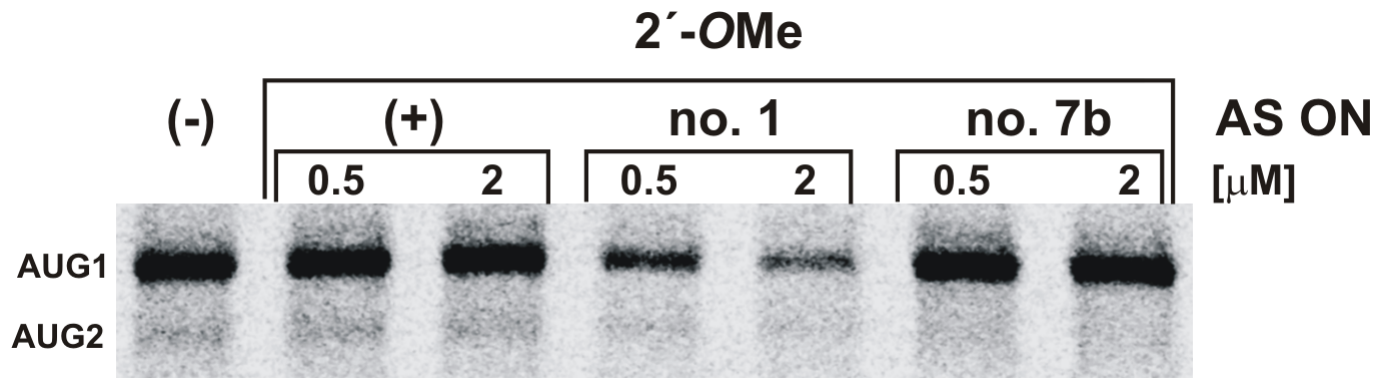
*In vitro* translation with 2′-*O*Me antisense oligomers no. 1 and no. 7b. The **a**utoradiogram shows the protein products of the translation reaction in RRL with the 5′-capped ΔNp53utr-Luc RNA in the presence of 2′-*O*Me antisense oligomers no. 1, no. 7b and the control oligomer (+), respectively. The control oligomer anneals to *Firefly* luciferase sequence. Lane (−) indicates control reaction with no antisense oligonucleotide addition.

Further, in order to test the impact of RNase H activity on protein synthesis 2′-*O*Me oligomers no. 1 and no. 7b were used in a translation assay in RRL. In the presence of 2′-*O*Me oligomer no. 1 the efficiency of protein synthesis from both initiation codons was reduced (Fig. 5), however, not as strongly as in the case of unmodified oligomer (see Fig. 3). Since 2′-*O*Me oligomers form heteroduplexes which are unable to recruit RNase H the 2′-*O*Me oligomer no. 1 might impair translation only *via* the steric-blocker action. Additionally, breakage of RNA in the G161-G168 region upon binding of oligomer no. 1 (see Fig. 4) might also influence the translation efficiency.

It turned out that 2′-*O*Me oligomer no. 7b caused quite a different effect compared with its unmodified variant (Fig. 5). It seemed that unmodified oligomer no. 7b triggered RNA truncation, which resulted from the RNase H activity in the lysate, and in consequence, the AUG2 initiation codon became more accessible for translation machinery. This was shown by an increased amount of *Renilla* luciferase represented by the lower band shown on the gel in Figure 3. However, inhibition of protein synthesis from AUG1 was modest taking into account a rather high activity of RNase H in RRL. More than 50% of radiolabelled RNA was cleaved in the presence of oligomer no. 7b in the same conditions (data not shown). The ratio between proteins obtained from both initiation codons was approximately 1∶1. This suggests that translation from AUG2 present in the truncated RNA might be preferred. On the contrary, 2′-*O*Me oligomer no. 7b decreased the translation occurring from AUG1 very slightly and no changes were observed for protein synthesis from AUG2.

Taken together, our results revealed that protein synthesis from both initiation codons for p53 and ΔNp53 could be altered differently by antisense oligonucleotides in rabbit reticulocyte lysate. The presence of 2′-*O*Me oligomer no. 1 or no. 7b probably disrupted the structure of the 5′-terminal region of p53 mRNA and/or interfered with mRNA scanning by the translation initiation complex. RNase H activity triggered by unmodified oligomers led to the generation of a truncated mRNA transcript which, in case of using oligomer no. 7b, was still a template for the translation machinery and protein synthesis from AUG2 became significantly more efficient.

### Down-regulation of p53 Expression Level in the Living Cell

A translation assay in RRL showed that antisense oligonucleotides were able to reduce protein synthesis from AUG1 for p53 (see Fig. 3 and Fig. 5). In order to find out whether this effect can be achieved in the living cell, MCF-7 cells were transfected with 2′-*O*Me oligonucleotides or their GAP variants and the level of endogenous p53 protein synthesis was analysed by western blots. It has been earlier shown that MCF-7 cells can be successfully transfected with antisense DNA oligomers [41].

**Figure 6:**
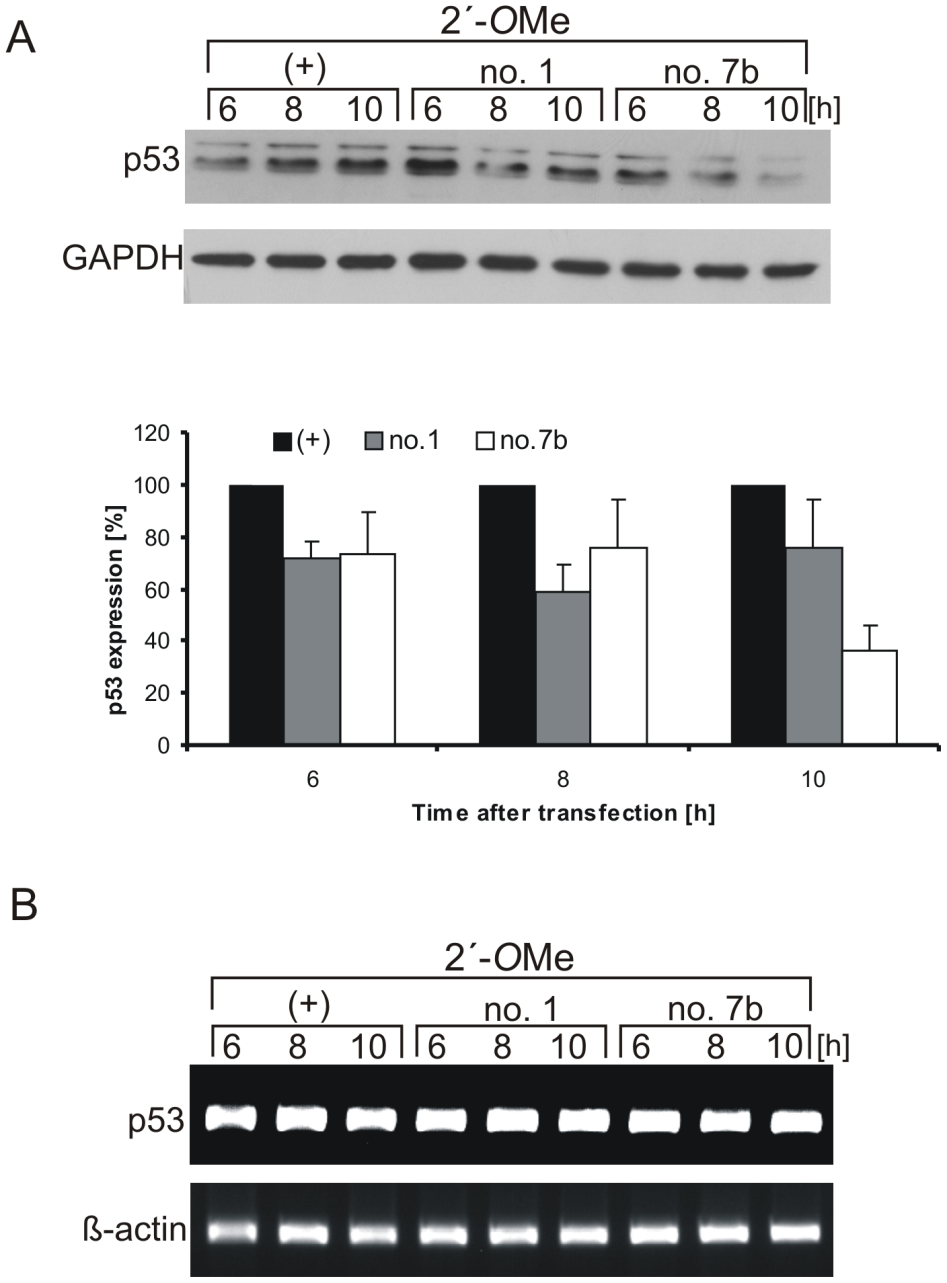
Disturbance of the p53 translation initiation process in MCF-7 cells in the presence of 2′-*O*Me antisense oligomers. (**A**) Cells were transfected with 2′-*O*Me oligomers: no. 1, no. 7b and control, respectively. Cells were harvested and lysed after specified time points and the level of endogenous p53 was determined by western blot using monoclonal antibody p53– Pab 1801. The GAPDH level was used to normalize the data. The p53 level in the presence of 2′-*O*Me oligomer no 1 or no. 7b was compared to the value of p53 protein level with control oligomer which was defined as 100%. The bar graph shows average and standard deviations for at least three independent experiments. (**B**) RT-PCR analysis of p53 mRNA and β-actin mRNA (as a control) extracted from the cell after the transfection of 2′-*O*Me oligomers at specified time points, respectively. The results from at least two independent experiments show no changes in the p53 mRNA level in the presence of 2′-*O*Me oligomers.

Both 2′-*O*Me oligomers no. 1 and no. 7b impaired p53 translation over a short time (Fig. 6A). Six hours after transfection the p53 expression was lower by approximately 30% compared with the level of p53 protein in the control sample in which 2′-*O*Me oligonucleotide complementary to *Firefly* luciferase sequence was used (Fig. 6A). Despite the lack of efficient inhibition of protein synthesis from AUG1 with 2′-*O*Me oligomer no. 7b in RRL (see Fig. 5), its effect *in vivo* was significant. Ten hours post transfection a further decrease in p53 expression, up to 40%, was observed. Twenty four hours after cell transfection with 1 µM 2′- *O*Me oligomer no. 7b more than 50% decrease of the p53 protein level was still observed (data not shown). The RT-PCR revealed no changes in the amount of p53 mRNA in the presence of 2′-*O*Me oligomers no. 1 or no. 7b (Fig. 6B) which confirmed that no RNase H-dependent mechanism was involved in down-regulation of the p53 protein level.

**Figure 7:**
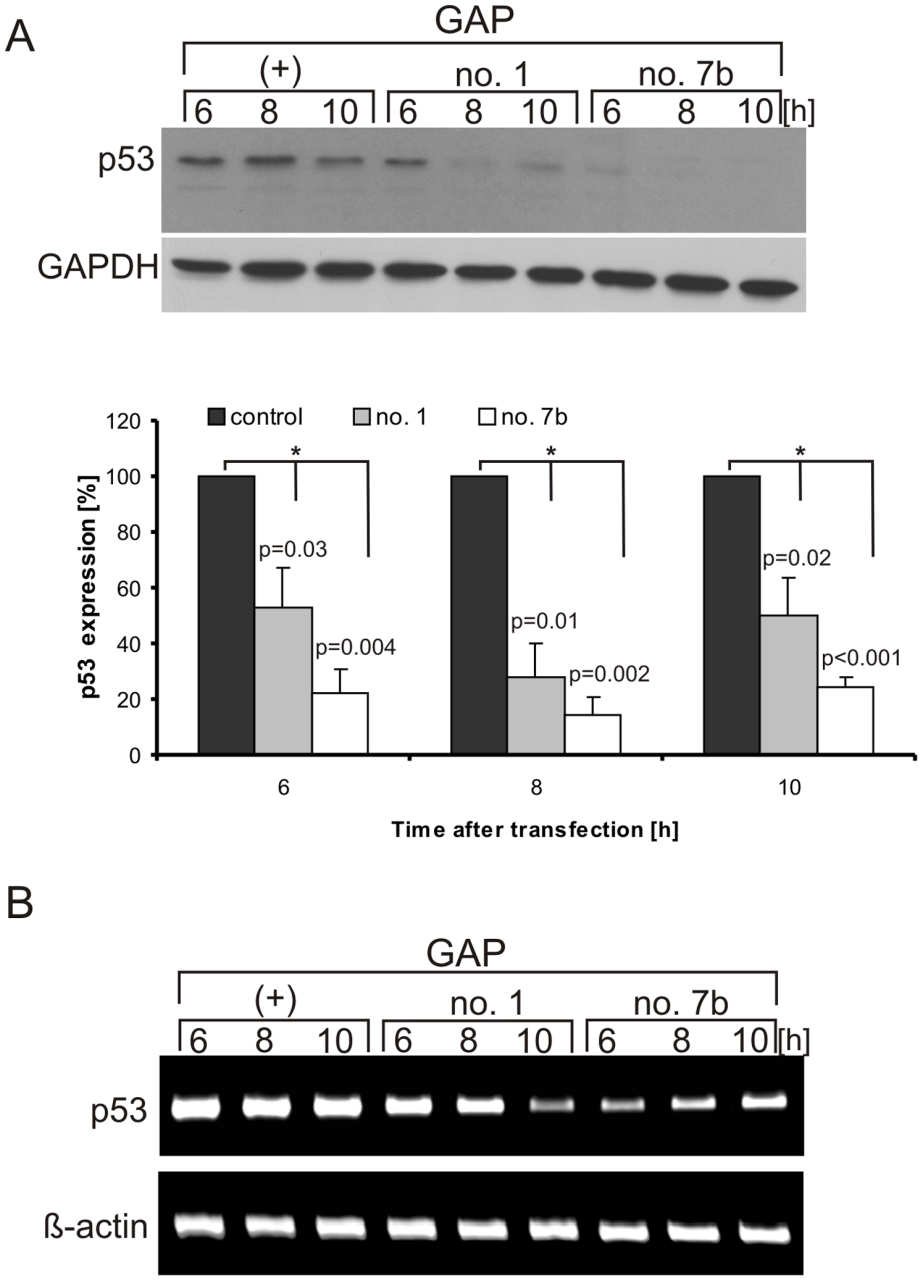
Down-regulation of p53 protein level in MCF-7 cells in the presence of GAP antisense oligomers. (**A**) Cells were transfected with GAP oligomers: no. 1, no. 7b and control, respectively. Cells were harvested and lysed after specified time points and the level of endogenous p53 was determined by western blot. The bar graph shows average and standard deviations for at least three independent experiments, while normalization was performed as described in the legend to Figure 6. (*) *p*-values were calculated using Student’s *t*-test (**B**) The RT-PCR analysis of p53 mRNA and β-actin mRNA (as a control) extracted from the cells after GAP oligomer transfection at specified time points, respectively. The data were obtained from at least two independent experiments.

Subsequently, GAP oligomers no. 1 and no. 7b which could induce RNase H activity were used in MCF-7 cells to test their effects on the p53 expression in the living cells. A strong reduction of the level of the p53 protein was observed for both gapmers (Fig. 7A). Inhibition of approximately 50% was achieved in the presence of GAP oligomer no. 1 and of 80% in the presence of GAP oligomer no. 7b. Moreover, both oligomers reduced the mRNA level compared with the control reaction (Fig. 7B) which was in line with the conclusion that RNase H activity was mainly responsible for changes in the amount of p53 protein.

**Figure 8:**
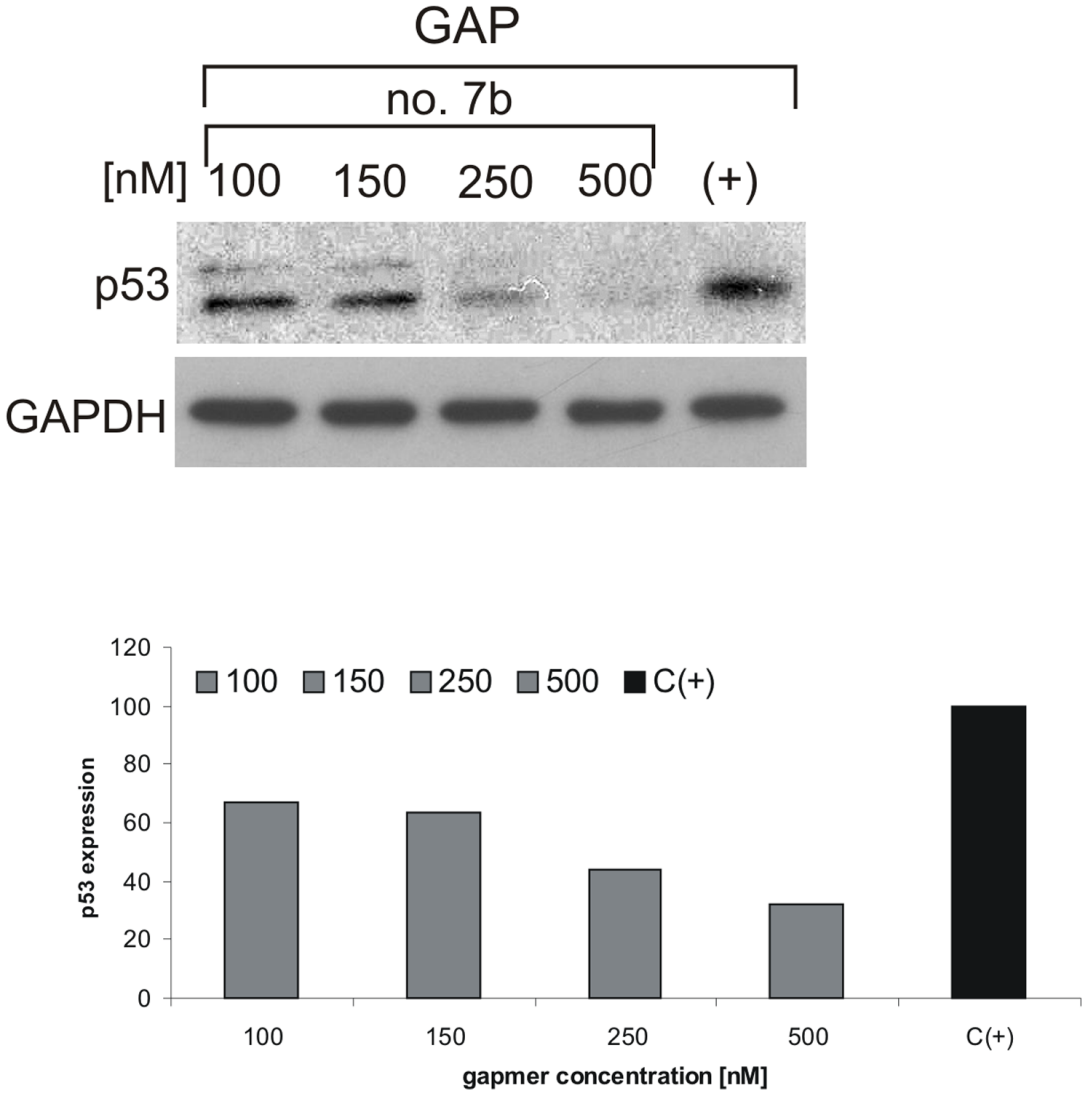
Inhibition of p53 expression in MCF-7 cells using different concentration of GAP oligomer no. 7b. The cells were transfected with GAP oligomer no. 7b with its final concentration of 0.5–0.1 µM and then 10 hours from transfection the cells were lysed and endogenous p53 was determined by western blot. The data was provided from at least two independent experiments.

Finally, GAP oligomer no. 7b was used in MCF-7 cells in various concentrations. It turned out that a decrease in p53 expression of approximately 40% can be reached over a short time, 10 hours post transfection, in the presence of this oligomer used at 100–150 nM concentration while with 500 nM oligomer inhibition of almost 70% was observed (Fig. 8).

## Discussion

The antisense oligonucleotide strategy was applied to modulate the expression of full-length p53 protein and its N-truncated ΔNp53 isoform. An analysis of accessibility of the 5′-terminal region of p53 mRNA to oligomer hybridization in a model ΔNp53utr-Luc transcript revealed the sites promising for translation inhibition in the lower part of helix G56-C169, at the 3′ side of helix U180-A218 and in the stretch spanning nucleotides in positions 219–228 (Fig. 1). Based on these results, a series of antisense oligonucleotides were designed and tested in an *in vitro* translation assay, in rabbit reticulocyte lysate (RRL). Oligomers no. 1 and no. 7b exhibited a strong impact on the translation occurring from both the initiation codons AUG1 and AUG2. Interestingly, in the presence of oligomer no. 7b the amount of protein synthetized from AUG2 was highly increased (Fig. 3).

Owing to the use of RRL to evaluate the efficiency of antisense oligonucleotides as translation inhibitors, additional experiments were performed to find out whether the RNase H activity remaining in the lysate might be involved in the observed effects. It has been reported that in RRL the RNase H activity is three times lower than in the wheat germ extract [36]. Moreover, to obtain effective arrest of translation in RRL the addition of exogenous RNase H enzyme was needed [37]. Nevertheless, the results of our translation reactions in the presence of antisense oligonucleotides suggested that the activity of RNase H in RRL might contribute to the observed effects (Fig. 4). In order to distinguish between the RNase H-dependent and independent mechanisms of translation inhibition 2′-*O*Me derivatives of selected oligomers were applied, which were unable to induce RNase H activity. It turned out that the unmodified oligomer no. 1 caused stronger inhibition of protein synthesis from AUG1 than its 2′-*O*Me variant. Most likely, this difference resulted from an additional RNase H support. On the other hand, unmodified oligomer no. 7b triggered RNase H enzyme and mRNA truncation in the region between AUG1 and AUG2. Unexpectedly, the truncated RNA was still used as a template for protein synthesis from the second initiation codon AUG2. A different impact on translation was observed in the presence of 2′-*O*Me oligomer no. 7b (Fig. 3 and Fig. 5). The oligomer did not considerably affect initiation from AUG1 and it had no influence on translation from AUG2. It is worthy of note that RRL is commonly used not only in the studies on protein synthesis inhibition but also in detailed analyses of the translation process. Our data show that the possible impact of RNase H activity remaining in RRL on translation results should always be taken into consideration in studies performed in that as well as other cell-free systems.

Strong enhancement of protein synthesis from the AUG2 initiation codon for ΔNp53, which occurred in the presence of unmodified oligomer no. 7b, is of potential interest (Fig. 3). It has been shown that ΔNp53 can oligomerize with p53 protein to reduce its total pool in the cell which results in the suppression of p53-mediated transcriptional activity [13]. Additionally, ΔNp53 seems to compete with p53 in the gene regulation due to the presence of DNA binding domain [5], [14]. Thus, modulation of ΔNp53 level might indirectly affect the p53 activity. We showed that antisense oligomer no. 7b induced RNase H enzyme activity and mRNA truncation in its 5′-terminal region. In a cell-free system in RRL the truncated RNA greatly contributed to the increased level of ΔNp53 isoform. However, in the living cell transcripts which were cleaved by RNase H would likely undergo rapid degradation and they would not serve as templates in translation [42], [43]. On the other hand, it has been reported that the initiation codons for p53 and ΔNp53 are located at the helix-bulge junctions [16]. We envisage that antisense oligonucleotides that alter the mRNA structure to expose the AUG2 codon or aptamers able to mimic the structural elements of the 5′-terminal region of p53 mRNA might be used to change the p53/ΔNp53 ratio in a controlled manner.

Recently, it has been demonstrated that a decrease in p53 protein level by antisense oligomers may be helpful as an additional treatment in radiotherapy of some solid malignant tumours to increase the radio-sensitivity of targeted cells [21]. Antisense oligonucleotides applied in our work down-regulated the efficiency of protein synthesis from AUG1 for p53 *in vitro*. To evaluate this effect in the living cell 2′-*O*Me oligomers and gapmers were transfected into MCF-7 cells [44], [45]. The suppression of p53 synthesis was observed for both types of oligomers (Fig. 6A and Fig. 7A). The hybridization site of 2′-*O*Me oligomer no. 1 located close to the AUG1 initiation codon could suggest that the inhibition of p53 synthesis resulted from the disturbance of the translation initiation process. Previously, it has been demonstrated that local structural changes in the G160-G167 region caused by silent mutations at third positions of codons 17, 18 and 19 of p53 mRNA influenced translation from AUG1 as well [17]. Stronger inhibition of p53 protein synthesis was observed with 2′-*O*Me oligomer no. 7b which was hybridized to nucleotides in positions 208–228. This region has been proposed as a potential binding site for Mdm2 protein. Moreover, it has been postulated that interaction of Mdm2 with the 5′-terminal region of p53 mRNA promotes p53 synthesis [46]. Therefore, we proposed that oligomer no. 7b might compete for the binding site with some *trans*-acting translation factors causing a decrease in p53 protein level.

It has been earlier shown that the antisense oligomer complementary to A130-G147 region that includes AUG1 in p53 mRNA was able to reduce p53 protein level by about 25–35% in irradiated non-small lung cancer cells (NSCLC). It has increased radiation-induced apoptosis of tumour cells [21]. In our studies GAP oligomer no. 7b, used at a similar concentration of 150 nM as applied previously, decreased the level of p53 protein in MCF-7 cells by approximately 40% over a short time post transfection. Stability of this oligomer in human serum was substantially increased by LNA modifications present at its 5′ and 3′ end. It has been shown that introduction of three LNA at each end of an oligonucleotide results in an approximately 10-fold increase of its half-life compared with the unmodified variant [47]. Taking into consideration the high effectiveness of GAP oligomer no. 7b in MCF-7 cells it seems to be a good candidate to be used as a support treatment in radiotherapy. However, for its practical applications further detailed research concerning the impact of this oligomer on the cell cycle and apoptosis induction in the irradiated tumour cells is necessary.

In our report, the potential applications of antisense oligonucleotides in the modulation of translation of p53 and ΔNp53 proteins were shown. The approaches based on antisense oligomers would shed more light on the way how the structural elements of the 5′-terminal region of p53 mRNA together with cellular proteins orchestrate to govern the translation initiation process. This is particularly attractive for studies performed in cells under stress conditions when a high increase of activity of p53 IRES is observed. Moreover, the antisense strategy can be potentially useful for the purpose of establishing an alternative medical treatment *via* regulation of p53 expression in some tumours.

## Supporting Information

Figure S1 **Structure probing of ΔNp53utr RNA.** (**A**) Autoradiograms show the products of Pb^2+^-induced cleavage analyzed by primer extension on 8% polyacrylamide gels in denaturing conditions. Guanosine residues are labelled on the left and the short and long run of the gel is shown. Lanes: (−) – control reaction in the absence of Pb^2+^ ions; A, T, C, G – sequencing reaction with adenosine, thymidine, cytosine and guanosine dideoxy terminating nucleotides, respectively. The cleavages induced in the presence of Pb^2+^ ions are displayed in panel D as black triangles. (**B**) Histogram of integrated and normalized DMS reactivity as a function of nucleotide position. Sites of modification were identified using the fluorescent-labelled DNA primer which anneals to a reporter protein coding sequence. The cDNA fragments were resolved by capillary electrophoresis. In order to create quantitative normalized SHAPE reactivity the raw data from capillary electrophoresis were analyzed using ShapeFinder software. Bars are coloured using the scale shown in panel D. (**C**) Normalized SHAPE reactivity as a function of nucleotide position. The nucleotides modified by DMS were determined by primer extension with a fluorescent-labelled DNA primer on a single capillary electrophoresis. Bars are coloured according to the scale shown in panel D. (**D**) The secondary structure model of ΔNp53utr RNA generated by RNAStructure 5.2 program by incorporation of the SHAPE reactivity as an energy function into the SHAPE-constrained algorithm. Nucleotide symbols are coloured according to their SHAPE reactivity. Nucleotide positions modified by DMS are denoted by circles and the cleavages induced by Pb^2+^ ions are displayed as black triangles.
(TIF)

Figure S2 **RNase H assay in the presence of antisense oligomer no. 1.** The RNase H assay on ΔNp53utr-Luc RNA with unmodified and GAP-modified oligomer no. 1 was performed. After 10 and 30 min incubation with RNase H from *E. coli*, RNA was phenol/chloroform purified and resolved on 8% polyacrylamide gel in denaturing conditions. Lanes: (−) – control reaction without antisense oligonucleotide, T1– limited hydrolysis by RNase T1. Selected guanosine residues are labelled on the left side of the autoradiogram.
(TIF)

Materials and Methods S1 **SHAPE Analysis, Pb^2+^-induced cleavage, DMS modification, RNase H assay.**
(DOC)
