# Supplementary figures and images for: Modulation of p53 Expression Using Antisense Oligonucleotides Complementary to the 5′-Terminal Region of p53 mRNA In Vitro and in the Living Cells
Source: PLoS One. 2013 Nov 11;8(11):e78863. doi: 10.1371/journal.pone.0078863 (PMC3824000; doi:10.1371/journal.pone.0078863)

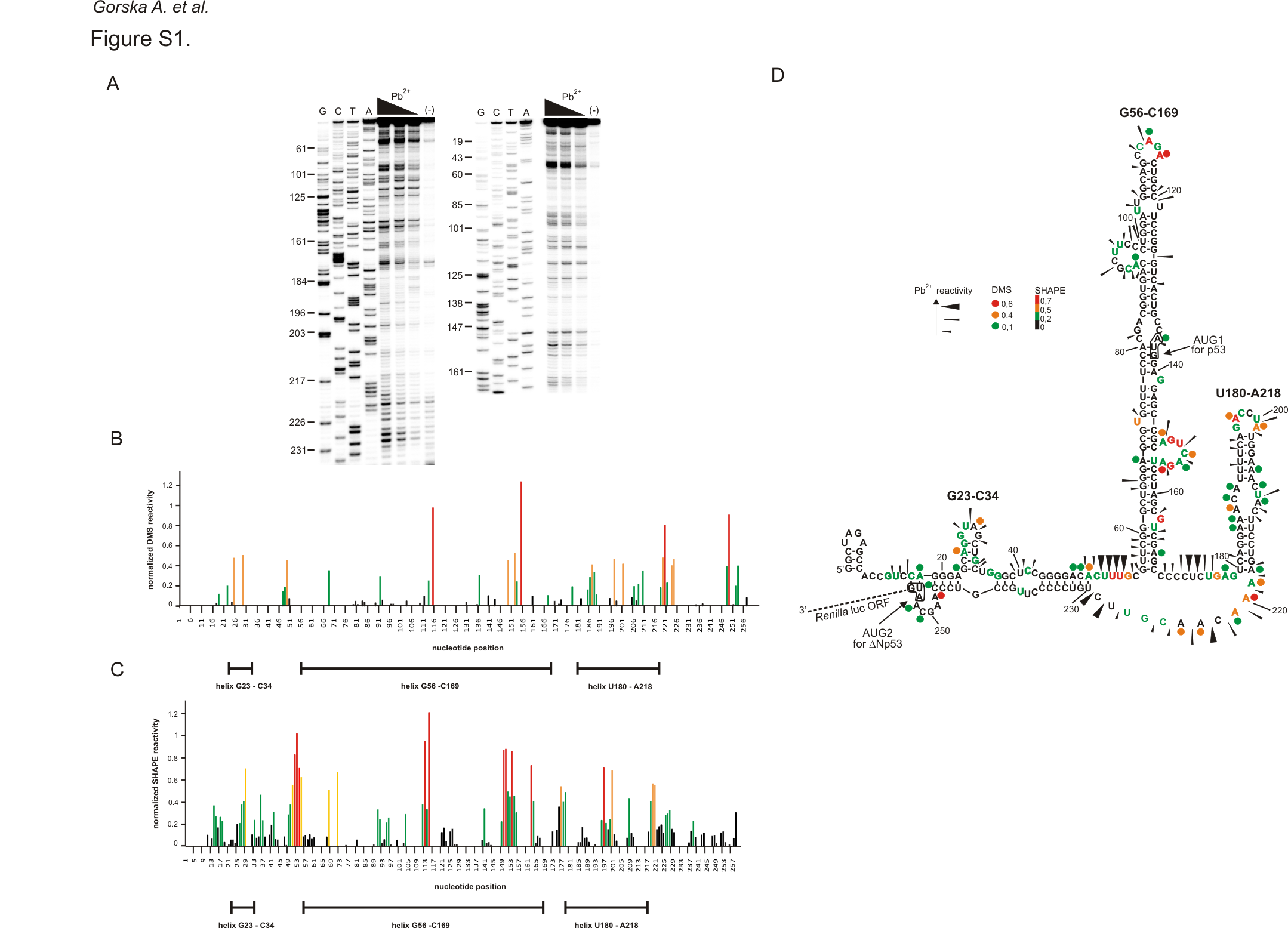

Supplement: Figure S1 — Structure probing of ΔNp53utr RNA. (A) Autoradiograms show the products of Pb2+-induced cleavage analyzed by primer extension on 8% polyacrylamide gels in denaturing conditions. Guanosine residues are labelled on the left and the short and long run of the gel is shown. Lanes: (−) – control reaction in the absence of Pb2+ ions; A, T, C, G – sequencing reaction with adenosine, thymidine, cytosine and guanosine dideoxy terminating nucleotides, respectively. The cleavages induced in the presence of Pb2+ ions are displayed in panel D as black triangles. (B) Histogram of integrated and normalized DMS reactivity as a function of nucleotide position. Sites of modification were identified using the fluorescent-labelled DNA primer which anneals to a reporter protein coding sequence. The cDNA fragments were resolved by capillary electrophoresis. In order to create quantitative normalized SHAPE reactivity the raw data from capillary electrophoresis were analyzed using ShapeFinder software. Bars are coloured using the scale shown in panel D. (C) Normalized SHAPE reactivity as a function of nucleotide position. The nucleotides modified by DMS were determined by primer extension with a fluorescent-labelled DNA primer on a single capillary electrophoresis. Bars are coloured according to the scale shown in panel D. (D) The secondary structure model of ΔNp53utr RNA generated by RNAStructure 5.2 program by incorporation of the SHAPE reactivity as an energy function into the SHAPE-constrained algorithm. Nucleotide symbols are coloured according to their SHAPE reactivity. Nucleotide positions modified by DMS are denoted by circles and the cleavages induced by Pb2+ ions are displayed as black triangles. (TIF) [file pone.0078863.s001.tif]

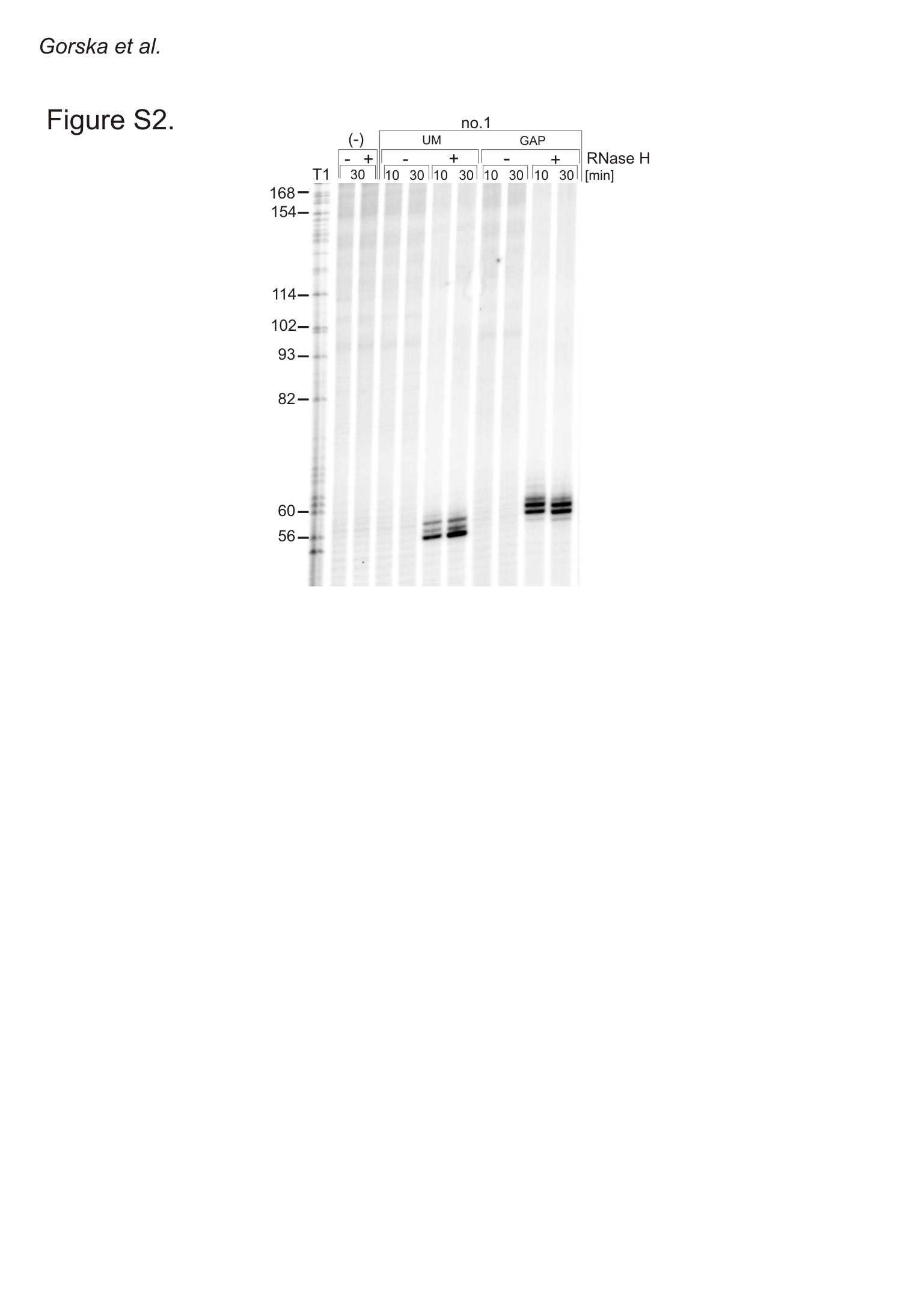

Supplement: Figure S2 — RNase H assay in the presence of antisense oligomer no. 1. The RNase H assay on ΔNp53utr-Luc RNA with unmodified and GAP-modified oligomer no. 1 was performed. After 10 and 30 min incubation with RNase H from E. coli, RNA was phenol/chloroform purified and resolved on 8% polyacrylamide gel in denaturing conditions. Lanes: (−) – control reaction without antisense oligonucleotide, T1– limited hydrolysis by RNase T1. Selected guanosine residues are labelled on the left side of the autoradiogram. (TIF) [file pone.0078863.s002.tif]
