# Supplementary material for: Modulation of p53 Expression Using Antisense Oligonucleotides Complementary to the 5′-Terminal Region of p53 mRNA In Vitro and in the Living Cells
Source: PLoS One. 2013 Nov 11;8(11):e78863. doi: 10.1371/journal.pone.0078863 (PMC3824000; doi:10.1371/journal.pone.0078863)
Supplement: Materials and Methods S1 — SHAPE Analysis, Pb2+-induced cleavage, DMS modification, RNase H assay. (DOC) [file pone.0078863.s003.doc]

**Materials and methods**

**SHAPE Analysis**

The reaction mixture containing: 20 pmol of ΔNp53utr-Luc RNA in renaturation buffer (10 mM Tris-HCl pH 8.0, 100 mM KCl, 0.1 mM EDTA) and in a final volume of 20 μl was heated at 90°C for 3 min and slowly cooled (0.1°C/sec) to 4°C. The folding buffer: 40 mM Tris-HCl pH 8.0, 5 mM MgCl_2_, 130 mM KCl, 0.1 mM EDTA was then added and water to the final volume of 146 μl. The sample was incubated at 37°C for 10 min and divided into two reactions. In the first reaction, the RNA solution was mixed with 7.3 μl of 55 mM *N*-methylisatoic anhydride (NMIA, Invitrogen) in DMSO (5 mM NMIA final concentration). The second control reaction contained DMSO without NMIA. Both reactions were incubated for 50 min at 37°C. The RNA was precipitated with 0.3 M sodium acetate pH 5.2, 1 μl of glycogen (20 mg/ml) and 3 volumes of ethanol. After centrifugation, RNA pellet was resuspended in 10 mM Tris pH 8.0 and 0.1 mM EDTA.

**Pb^2+^-induced cleavage**

Prior to cleavage reaction with Pb^2+^ ions, 130 pmol of unlabelled ΔNp53utr-Luc RNA was renatured in the buffer: 40 mM NaCl, 10 mM Tris-HCl pH 7.3, 10 mM MgCl_2_ by heating for 5 min at 65°C and slowly cooling to 37°C. Subsequently, the RNA solution was partitioned into four samples and the lead acetate solution was added to the final concentration of 0.25 mM, 0.5 mM and 1 mM, and an equal volume of water was added to a control reaction. After incubation at 37°C for 3 min, the reactions were terminated by mixing their aliquots with 8 M urea/dyes/20 mM EDTA solution.

**DMS modification**

10 pmol of ΔNp53utr-Luc RNA in 100 mM NaCl in a final volume of 160 μl was renatured for 3 min at 90°C and then slowly cooled (0.1°C/sec) to 4°C. The folding buffer (50 mM HEPES pH 8.0, 5 mM MgCl_2_, 100 mM NaCl) was added and the reaction mixture was incubated at 37°C for 10 min. The RNA solution was mixed with 10 μl of 10% DMS in ethanol (0.25% final concentration) and incubated at 37°C for 3 min. Additional control reaction was performed to check the efficiency of DMS inactivation. The reactions were placed on ice and the RNA was precipitated with 0.3 M sodium acetate pH 5.2, 1μl of glycogen (20 mg/ml) and 3 volumes of ethanol. After centrifugation, the RNA was resuspended in RNase-free water.

**RNase H assay**

2.5 pmol of 5′-end-[^32^P]-labelled ΔNp53utr-Luc RNA was renatured in the buffer containing: 40 mM Tris-HCl pH 8.0, 40 mM KCl, 10 mM MgCl_2_, 1 mM DTT and 0.1 mM EDTA by heating for 2 min at 65°C and slow cooling to 37°C. Subsequently, *E. coli* RNase H was added to the final concentration of 250 units/ml. The control reaction was also carried out in the absence of RNase H. The cleavage reactions were induced by adding antisense oligomer no. 1 to the final concentration of 2 μM. After 10 and 30 min incubation at 37°C the reactions were stopped by addition of EDTA (100 mM final concentration) and supplemented with water to the final volume of 100 μl. The reaction products were purified by phenol/chloroform (1:1, v/v) extraction and precipitated with 0.3 M sodium acetate pH 5.2, 1 μl of glycogen (20 mg/ml) and 3 volumes of ethanol at –20°C overnight. After centrifugation, the pellets were resuspended in 10 μl of RNase-free water.
